# A modular semantic-structural pipeline for visual decoding from primate spiking data via selective temporal integration

**DOI:** 10.1162/IMAG.a.1299

**Published:** 2026-07-13

**Authors:** Matteo Ciferri, Matteo Ferrante, Nicola Toschi

**Affiliations:** Department of Biomedicine and Prevention, University of Rome Tor Vergata, Rome, Italy; Tether Evo, Edificio Centro Corporativo Presidente Plaza, San Salvador, El Salvador; A.A. Martinos Center for Biomedical Imaging, Harvard Medical School/MGH, Boston, MA, United States

**Keywords:** visual decoding, intracortical recordings, primate visual cortex, machine learning, generative reconstruction, brain–computer interfaces

## Abstract

Characterizing the information content of intracortical signals during visual processing is a central challenge in systems neuroscience. We address the problem of decoding visual information from high-density intracortical recordings in primates, using the THINGS Ventral Stream Spiking Dataset. We systematically evaluate the effects of model architecture, training objectives, and data scaling on decoding performance. Results show that decoding accuracy is jointly driven by non-linearity and selective temporal aggregation, rather than heavier sequence modelling in this data regime. A simple model combining temporal attention with a shallow MLP achieves up to 70% top-1 image retrieval accuracy, outperforming linear baselines as well as recurrent and convolutional approaches. Scaling analyses reveal predictable diminishing returns with increasing input dimensionality and dataset size. Building on these findings, we design a modular generative decoding pipeline that combines low-resolution latent reconstruction with semantically conditioned diffusion, generating plausible images from 200 ms of brain activity. This framework provides principles for brain-computer interfaces and semantic neural decoding.

## 1 Introduction

A complete account of perception and behavior must bridge neural representations with mental states, linking spikes and field potentials to the contents of subjective experience and overt action. Recent progress in cognitive science and computational neuroscience has been catalyzed by three intertwined developments. First, community-driven efforts now release large, meticulously curated datasets that pair rich sensory stimulation with high-resolution neural recordings (Allen et al., 2022; Chang et al., 2019; Hebart et al., 2023; Horikawa & Kamitani, 2017). Second, advances in machine learning—particularly deep generative modeling and scalable optimization—provide expressive function classes capable of capturing the complex structure of brain-world mappings (Antonello et al., 2023; Banville et al., 2025; Oota et al., 2023). Third, experimental practice is changing from *wide* surveys of many individuals to *deep*, longitudinal studies that expose a few subjects to tens of thousands of stimuli, drastically increasing statistical power (Kupers et al., 2024).

These factors have revived bidirectional modeling of the stimulus–brain relationship. *Encoding* models predict neural responses from sensory features, helping us understand the functional organization of the cortex, whereas *decoding* models seek to reconstruct stimuli—or latent variables relevant to the task—from brain activity, a line of work central to basic science, as well as emerging brain–computer interfaces. Successes span multiple modalities (EEG, MEG, fMRI, ECoG, and Utah array recordings) and cognitive domains, including language comprehension, speech production, music, and vision (Bazeille et al., 2021; Oota et al., 2023). However, even with invasive data, key questions persist: What properties of intracortical spike trains carry the information necessary for high-fidelity decoding? How do architectural choices—linear versus nonlinear models, temporal aggregation windows, loss functions—shape performance limits? And how do these factors interact with scale, both in terms of training data and in terms of the dimensionality of neural input?

**Fig. 1.**
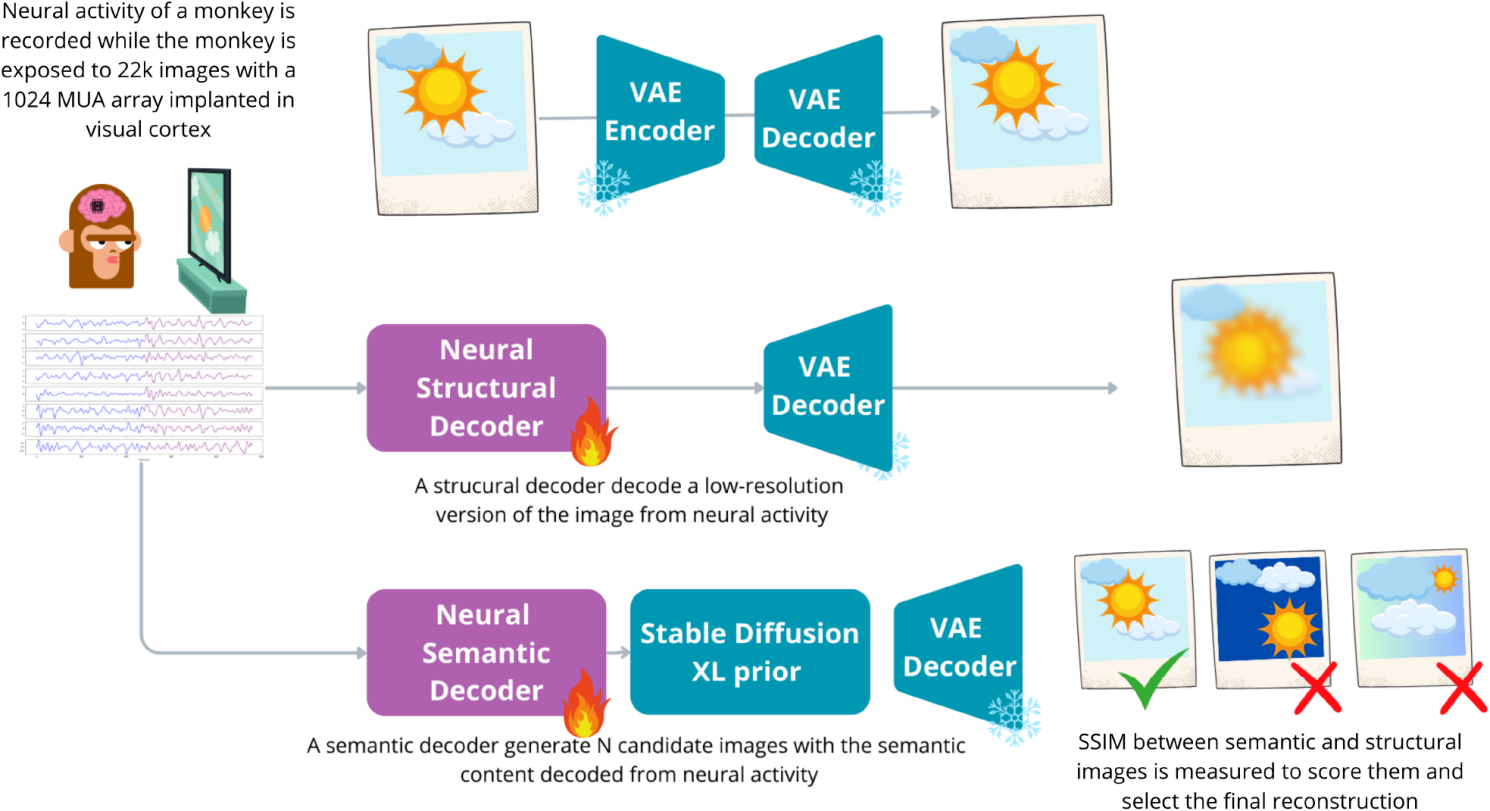
Overview of our generative decoding framework. Neural activity is recorded from a macaque implanted with a 1,024-channel MUA array while viewing around 22 k natural images. A two-branch decoding pipeline is used: (top) a **structural decoder** maps neural signals to a low-resolution latent representation using a VAE decoder, providing a faithful but coarse reconstruction; (bottom) a **semantic decoder** generates multiple candidate images via Stable Diffusion XL conditioned on semantic information decoded from the same neural signal. The final image is selected by computing the SSIM between each candidate and the structural image, effectively combining the structural accuracy of the low-res decoder with the generative power of the semantic branch.

Our study leverages the recently released THINGS Ventral-Stream Spiking Dataset (TVSD) (Papale et al., 2025), in which two macaques viewed more than ∼20 *k* natural images while they recorded multi-unit activity (MUA) from ∼2,000 channels distributed across V1, V4 and IT at 30 kHz. Each image in the training partition was shown once, while 100 held-out images were repeated 30 times to boost signal-to-noise ratio and enable stringent cross-validation. In this work, we tackle the following research questions:

1. **Temporal versus architectural complexity.** What role does temporal structure play in neural decoding, and what types of models are best suited to capture it? To explore this, we trained various models to decode neural data into semantic image representations obtained using a frozen CLIP model. We systematically compared architectures ranging from simple linear models to recurrent neural networks capable of capturing complex nonlinear dynamics. We show that two factors jointly drive decoding performance: the introduction of nonlinearity and selective temporal integration. The former produces the larger absolute gain, while the latter provides a consistent and reliable improvement on top of it. Importantly, heavy sequence models such as LSTMs and TCNs do not outperform our lightweight attention mechanism, suggesting that modeling complex temporal dependencies end-to-end is less effective than selective integration in this data regime. The evaluation was carried out by measuring the accuracy of image retrieval on held out test data, specifically quantifying the model’s ability to identify exact images from neural activity using top-1 and top-5 retrieval metrics.
2. **Objective functions.** We compare two ways of predicting vector representations from brain activity: mean squared error loss and representation alignment with contrastive learning. Similarly to the previous point, we used retrieval performance as a probe of the quality of decoded embeddings.
3. **Scaling laws.** We chart the performance of our best model in the retrieval task as a function of (i) the number of trials and (ii) the number of principal components derived from neural channels, revealing predictable regimes of diminishing returns that inform experimental design.
4. **Generative Decoding.** We introduce a two-stage decoder that samples candidate images from a frozen generative prior (Stable Diffusion from Podell et al., 2023) conditioned on brain-predicted CLIP representations, and performs rejection sampling guided by structural similarity to a low-resolution neural reconstruction, achieving near-photorealistic reconstructions from ≈200 ms windows of activity. See Figure 1 for an overview of our generative decoding pipeline.

To address the first two research questions, we used zero-shot image retrieval as a proxy for assessing the quality of the mapping between brain activity and semantic visual representations. Crucially, we avoid generative models at this stage to minimize the confounding influence of strong image priors. When a generative model produces a high-quality image, it becomes difficult to discern whether the result reflects successful decoding or simply the model’s inherent ability to produce photorealistic samples (Shirakawa et al., 2024). Retrieval-based evaluation offers a more transparent and interpretable benchmark: the model must identify the correct image from a fixed candidate pool based solely on the neural signal, allowing precise measurement of top-1 and top-5 accuracy.

However, retrieval has its limitations: mainly, its reliance on a predefined candidate set, which restricts generalization. For this reason, once we validated that our model achieved strong performance in this constrained setting, we turned to the more ambitious goal of generative decoding, confident that our brain to embedding map is robust due to prior validation in a retrieval setting. We propose a framework to extend brain-to-image mapping beyond fixed image sets, enabling open-ended visual reconstruction that overcomes the limitations of retrieval-only evaluation.

Together, these results introduce a practical decoding framework rather than a task-specific demo: (i) a lightweight temporal selection mechanism that turns noisy, high-rate intracortical activity into a stable semantic representation, and (ii) a modular retrieval-generation pipeline that operationally separates semantic identity from structural layout. This division makes the approach auditable, computationally efficient, and compatible with future closed-loop brain–computer interfaces.

The present work pursues two related but distinct aims. The primary aim is computational: we systematically compare decoding architectures, training objectives, and scaling regimes to identify what drives performance when mapping intracortical MUA to visual representations. The secondary aim is exploratory and neuroscientific: we use the structure of decoding performance as a probe of the information available in ventral stream activity. In particular, we extend our approach as a window into neural coding principles, including regional specialization across V1, V4, and IT, and the temporal dynamics of visual processing. We adopt CLIP embeddings for decoding targets as a computational convenience, not as a theoretical commitment to a representationalist account of neural coding.

### 1.1 Related works

Recent years have seen great advances in decoding visual (and other) stimuli from neural activity, primarily in non-invasive settings such as fMRI (Antonello et al., 2023; Bazeille et al., 2021; Ciferri et al., 2025; Ferrante, Boccato, Ozcelik, et al., 2024; Gallant et al., 2012; Huth et al., 2016; Oota et al., 2023). Methods using pre-trained vision language models such as CLIP, paired with linear regression or contrastive learning, have enabled retrieval-based decoding and increasingly realistic image reconstruction when combined with diffusion models (Chen et al., 2023; Ferrante, Boccato, Passamonti, & Toschi, 2024; Lin et al., 2022; Ozcelik & VanRullen, 2023; P. Scotti et al., 2023; Xia et al., 2024).

A relevant line of work in the fMRI reconstruction literature has established a conceptual template that combines semantic and structural decoding streams. Takagi and Nishimoto (2023) decode a high-level CLIP embedding from fMRI and use it to condition a latent diffusion model, while separately reconstructing low-frequency spatial structure from brain activity to guide the generation. Similarly, MindEye2 (P. S. Scotti et al., 2024) align fMRI representations to CLIP space via contrastive learning and combine them with diffusion priors for image reconstruction. At a high level, our pipeline shares this two-stream structure: we decode a semantic representation from neural activity and combine it with a coarse structural estimate to guide image generation. However, our approach differs from these frameworks along several dimensions that are consequential for the present setting. First, our input modality is high-density intracortical MUA from macaque visual cortex (the TVSD dataset by Papale et al., 2025), which presents a fundamentally different signal regime: high temporal resolution (30 kHz, 200 ms windows) and high channel count (1,024) compared to the fMRI datasets used in prior work, which offer thousands of repetitions per subject but no millisecond-scale temporal structure. This motivates our core architectural choice: a soft temporal attention mechanism that learns to selectively weight individual milliseconds of activity, rather than treating the neural signal as a static spatial pattern. Second, our candidate selection strategy differs from prior work: rather than using a fixed or learned prior to guide generation, we employ rejection sampling based on structural similarity (SSIM) between generated candidates and a low-resolution VAE reconstruction decoded from brain activity. This mechanism is training-free at generation time and explicitly decouples the semantic and structural objectives, making the pipeline modular and interpretable. Third, our evaluation is grounded in a retrieval-based benchmark using frozen CLIP embeddings before any generative model is applied, which avoids the confound of strong generative priors inflating perceived reconstruction quality, a concern explicitly raised by Shirakawa et al. (2024).

The most closely related to our work is the MonkeySee study by Le et al. (2024), which also uses TVSD and proposes a CNN-based end-to-end decoder with a learned inverse retinotopic mapping module to reconstruct images from neural signals. Their model emphasizes pixel-level realism and spatial interpretability, incorporating adversarial training and VGG feature losses to produce high-fidelity images. In contrast, we focus on mapping between brain activity and semantic representations using zero-shot retrieval with frozen CLIP embeddings, sidestepping the confounding of strong generative priors. This setup allows us to systematically assess how model class (linear vs. nonlinear), temporal structure, and scaling of training set size impact decoding performance. Only after validating our brain-to-embedding map in this controlled setting, do we extend it to open-ended generative decoding using rejection sampling.

## 2 Material and Methods

**Fig. 2.**
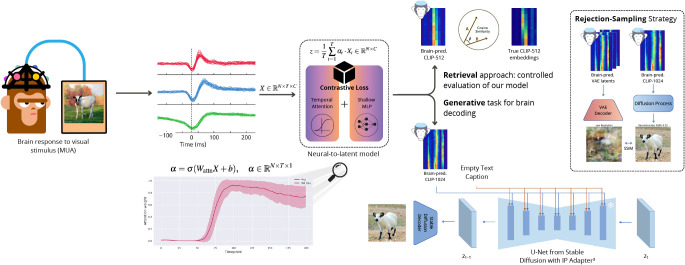
Overview of the proposed neural-to-semantic visual decoding pipeline. Multielectrode MUA responses evoked by visual stimuli are preprocessed and fed into a temporal-attention module that learns stimulus-dependent weighting over time, followed by a shallow MLP that maps neural activity into the CLIP embedding space. Training is supervised through a contrastive loss that aligns predicted neural embeddings with ground-truth CLIP representations of the viewed images. At test time, the predicted embeddings support two complementary tasks: (i) retrieval, where the model selects the most semantically similar image from a candidate set, and (ii) generation, where the embedding conditions a Stable Diffusion model (via an IP-Adapter) to synthesize novel images preserving the semantics inferred from neural activity. A rejection-sampling strategy ensures that generated samples remain structurally consistent with the original ones.

We propose a brain decoding framework to estimate the embedding of a visual stimulus directly from neural recordings (Fig. 2). The goal is to reconstruct a meaningful representation of the perceived image from intracortical signals, allowing two downstream applications: (i) *stimulus retrieval*, where the estimated embedding is compared against a set of candidate stimuli, and (ii) *image generation*, where the predicted embedding is used as input to a generative model to synthesize a recognizable version of the original visual input.

### 2.1 Ethics statement

This work is based exclusively on computational analyses of the publicly available THINGS Ventral Stream Spiking Dataset. No new animal experiments were conducted as part of this study. The original electrophysiological recordings were obtained from non-human primates by the dataset authors.

### 2.2 Data

We conduct our analysis on both **Monkey F** and **Monkey N**, from the THINGS Ventral Stream Spiking Dataset (TVSD) (Papale et al., 2025). This dataset comprises intracortical multi-unit activity (MUA) recorded from 15 Utah arrays implanted in visual areas V1, V4, and IT of two macaque monkeys. Neural responses were collected while the monkeys passively viewed more than 20 k unique natural images from the THINGS database, covering a broad distribution of object categories. Each image was presented once for 200 ms, interleaved with 200 ms of a gray screen. The recordings were sampled at 30 kHz and temporally aligned with stimulus onset. For decoding, we used a 200 ms post-stimulus window. A subset of 100 images was presented 30 times each and held out entirely for testing. Critically, these 100 images were never included in the training or validation sets, ensuring strict image-level separation with no leakage. The remaining images were used for training, with 10% held out as validation set for hyperparameter optimization. This design enables both training on a large-scale dataset and reliable, low-noise testing. The neural data were preprocessed as follows. Let $X_{\text{raw}}\;\in {\mathbb{R}}^{N\times T\times C}$ denote the raw MUA data, with *N* the number of samples, *T* = 200 timepoints, and *C* = 1,024 channels. The neural data were standardized by z-scoring each channel across all timepoints and samples in the training set. The same normalization parameters were then applied to the test set.

### 2.3 Neural model

In this section, we describe our proposed decoding model. The target representations $Y\in {\mathbb{R}}^{N\times D}$ , with *D* = 512, consist of high-level visual embeddings corresponding to the presented images, computed from the pretrained CLIP visual encoder (Radford et al., 2021).

In order to learn a mapping from MUA signals to the corresponding image embedding, we propose a neural architecture that can take into account the time evolution of the neural response. The model is designed to attend over the temporal dimension of the neural sequence and project the aggregated representation to the 512-dimensional target space.

Given an input tensor $X\in {\mathbb{R}}^{\text{N}\times T\times C}$ , the model computes a soft attention over time points:

$$\alpha =\sigma \left(W_{\text{attn}}X+b\right),\;\alpha \in {\mathbb{R}}^{N\times T\times 1}$$

where $W_{\text{attn}}$ denotes a linear layer and σ is the activation of the sigmoid. The attended representation is computed as:

$$z=\frac{1}{T}\sum_{t=1}^{T}\alpha_{t}\;\cdot X_{t}\in {\mathbb{R}}^{N\times C}$$

The vector *z* is then projected into the output space via a multilayer perceptron consisting of two fully connected layers with GELU activation and dropout:

$$\hat{Y}=W_{2}\cdot \;\;\text{Dropout}\left(\text{GELU}\left(W_{1}z+b_{1}\right)\right)+b_{2},\;\hat{Y}\in {\mathbb{R}}^{N\times D}$$

The training objective is a *contrastive loss* based on cosine similarity between predicted and ground-truth embeddings. Let $S\in {\mathbb{R}}^{N\times N}$ be the cosine similarity matrix between $\hat{Y}$ predicted outputs and *Y* targets: $S_{ij}=\frac{{\hat{y}}_{i}^{⊤}y_{j}}{∥{\hat{y}}_{i}∥∥y_{j}∥}.$ The loss function is a variant of the NT-Xent loss with temperature scaling τ, learned during training:

$${ℒ}_{\text{contrastive}}=-\frac{1}{N}\sum_{i=1}^{N}\text{log}\left(\frac{\text{exp}\left(S_{ii}/\tau \right)}{\sum_{j=1}^{N}\text{exp}\left(S_{ii}/\tau \right)}\right)$$

In order to contextualize the performance of our proposed model, we compare it against several standard baselines commonly used in brain decoding literature. The following baseline models were considered:

- Linear Model with Temporal Attention: similar to the proposed model, this variant uses the same temporal attention mechanism, but replaces the final MLP with a single linear layer to project the representation to the CLIP embedding space.
- Linear Model with Temporal Averaging: a linear regression trained on neural features obtained by averaging the neural signal over the temporal dimension, that is, reducing each trial from ℝ*^T^*^×^*^C^* to ℝ*^C^*.
- Linear (or MLP) Model on Flattened Input: a mapping trained on the fully flattened MUA signal, reshaped from ℝ*^T^*^×^*^C^* to a 1D-vector ℝ*^T^*^·^*^C^*.
- MLP with Temporal Averaging: a feedforward neural network trained on the same time-averaged representation as above, introducing non-linearity over the input features at channel-wise level.
- Recurrent Neural Network: an LSTM processes the neural sequence over time. The hidden state of the LSTM is extracted and passed through a projection layer to obtain the predicted embedding.
- Temporal Convolutional Network: a model composed of stacked 1D convolutional layers, followed by adaptive pooling. The representation is then passed through an MLP to produce the final embedding.

Hyperparameters, including learning rate, batch size, network depth for deep models, and regularization strength, were selected based on the performance of the validation set. See Figure S3 and Table S7 in Supplementary Material for completeness. The models were trained with both contrastive learning and standard regression objectives. All experiments were carried out on a high performance server equipped with eight NVIDIA A100 GPUs (80 GB each, interconnected via NVLINK), 256 CPU threads, and 2 TB of system memory.

### 2.4 Task 1: Stimulus retrieval

In order to assess the quality of the predicted embeddings, we performed a retrieval task, where each predicted embedding yˆ *_i_* from the test set is matched against all ground truth embeddings ${\left\{y_{j}\right\}}_{j=1}^{N}$, and the nearest neighbors are retrieved based on cosine distance. After generating predictions for all test samples in evaluation mode, we collected both the predicted embeddings $\hat{Y}\in {\mathbb{R}}^{N\times D}$ and the corresponding ground truth embeddings $Y\in {\mathbb{R}}^{N\times D}$ . The cosine distance was used to compute nearest neighbors in the embedding space: $\text{dist}\left({\hat{y}}_{i},y_{j}\right)=1-S_{ij}=1-\frac{{\hat{y}}_{i}^{⊤}y_{j}}{∥{\hat{y}}_{i}∥∥y_{j}∥}.$ For each test sample *i*, we identified the top- *k* most similar ground-truth embeddings over the test set. We computed the proportion of test samples for which the nearest neighbor is the ground truth target embedding, that is, when the index of the closest neighbor matches the sample index (Top-1 Accuracy). We also evaluated the proportion of test samples for which the ground-truth embedding appears within the top-5 retrieved neighbors (denoted as Top-5 Accuracy). This retrieval setup provides a quantitative measure of the semantic similarity between predicted and target embeddings, and acts as an indirect proxy of brain to image representation mapping quality in form of a decoding metric. We also report the retrieval results for each specific ROI in the visual areas V1, V4, and IT.

### 2.5 Scaling laws

In order to investigate how the performance of the decoding model scales with different properties of the input data, we conducted two different sets of controlled experiments evaluating the impact of (i) the input dimensionality, and (ii) the size of the training set (i.e., number of available samples). We applied Principal Component Analysis (PCA) to the MUA signals across channels to analyze input dimensionality impact on performance. We fitted PCA models (on trial-time samples) with different components, and projected both the training and test sets into the reduced channel subspace. The resulting PCA-reduced data had shape ${\mathbb{R}}^{N\times T\times C^{\prime }}$, where *C*^′^ < *C* is the selected number of components. This allowed us to test the model under different values of *C*^′^ while keeping the temporal resolution constant. To evaluate the effect of training data size on model performance, we subsampled the training set in a different scenario using a random selection of *N*′ samples, with *N*′ < *N*. Specifically, we randomly selected a fixed number of training samples, using a controlled random seed for reproducibility. The test set remained fixed across all experimental conditions. For each experimental condition—defined by a particular number of PCA components or training samples—we trained a new instance of the neural model from scratch and evaluated it on the full test set. This procedure enabled us to characterize the scaling behavior of the model under varying data availability and spatial resolution, highlighting how model performance is affected by data constraints commonly encountered in neural decoding settings.

### 2.6 Task 2: Image generation

In addition to retrieval-based evaluation, we explore a generative setting in which the predicted neural embeddings are used to synthesize images. The goal of this task is to demonstrate that the decoded representations retain sufficient visual semantics to condition a generative model and reconstruct a plausible version of the original stimulus. We use the Stable Diffusion model as the generative backbone (Podell et al., 2023). In order to condition the model on high-level visual embeddings, we incorporate the IP-Adapter module (Ye et al., 2023) into the diffusion pipeline. This adapter enables conditioning via learned visual representations rather than textual prompts. We first train our neural model with output dimensionality matching that of the IP-Adapter input (e.g., 1,280) to predict visual embeddings from MUA data. In a second setup, we train the same model to directly predict the flattened latent representation of the image expected by the Stable Diffusion VAE (a tensor of shape 4×32×32 , i.e., 4,096 dimensions). While the latents are decoded through the frozen VAE to obtain a low-resolution (256 × 256) structural preview of the stimulus, the embeddings are passed to the IP-Adapter consistently with classifier-free guidance. The IP-Adapter scale is set to 0.6; guidance scale to 10.

Our approach is inspired by the recent trend of increasing test-time computation in AI systems (Damian et al., 2022; Ji et al., 2025). Leveraging the strong mapping between brain activity and semantic content, and the ability of generative models to reconstruct semantically coherent images, we first generate *N* candidate semantic images for each trial. All candidates are resized to 256 × 256 and smoothed with a Gaussian blur (σ = 6 pixels) to suppress high-frequency texture noise before structural comparison. SSIM (Wang et al., 2004) is then computed between each smoothed candidate and the low-resolution VAE preview. The candidate with the highest SSIM score is selected as the final reconstruction. The number of candidates (N = 10) was chosen based on the ablation reported in the Supplementary Material (Fig. S1), where performance gains plateau beyond approximately 10 candidates for most metrics.

## 3 Results

### 3.1 Retrieval accuracy

**Fig. 3.**
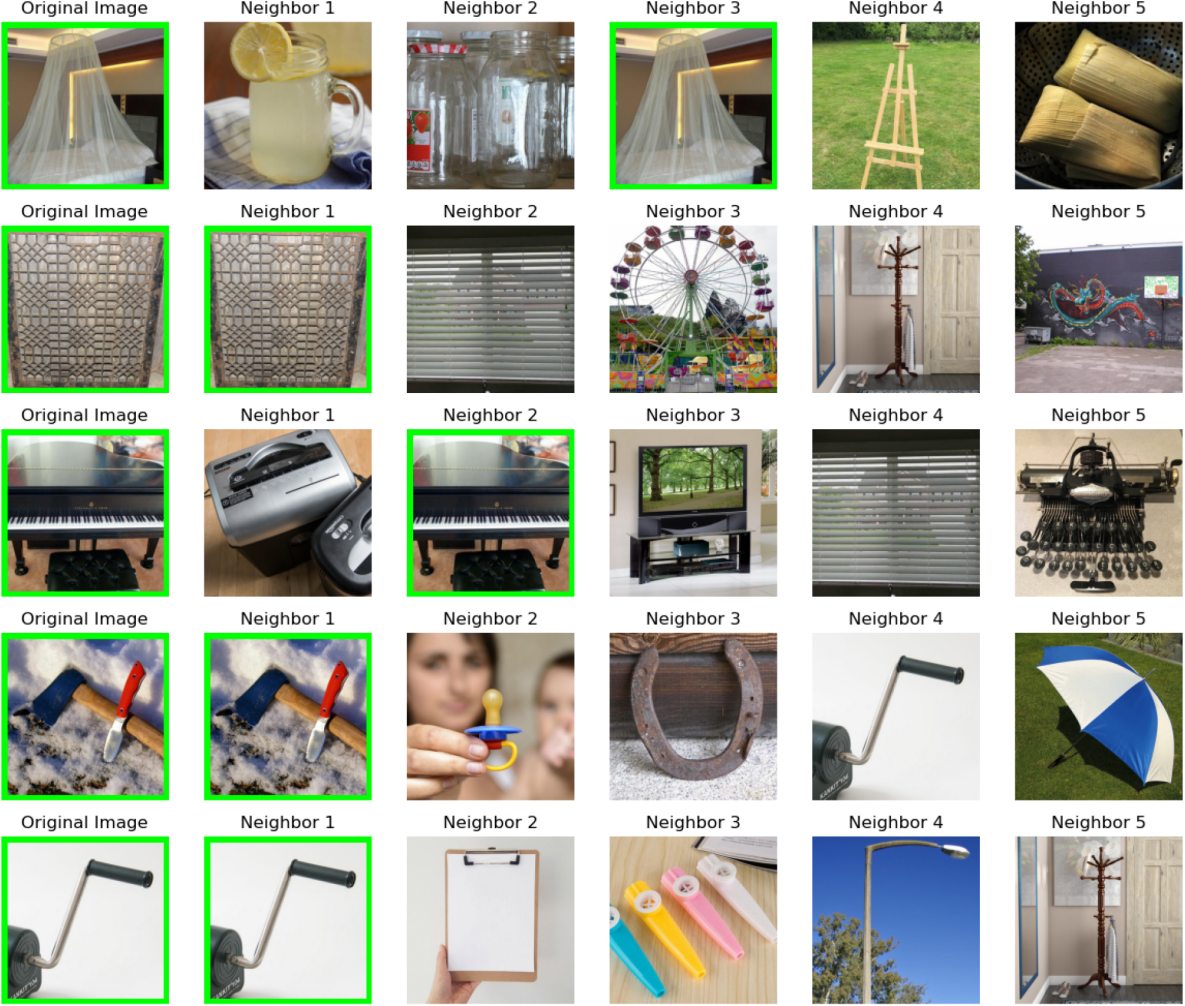
Top-5 image retrieval examples based on predicted embeddings. Each row shows one test sample: the original image (left) and the five nearest neighbors retrieved from the test set.

We evaluated the decoding performance using a retrieval task in which the predicted visual embeddings were matched against the ground truth embeddings of all test samples. The model was considered successful if the correct target appeared within the top-*k* nearest neighbors, with Top-1 and Top-5 accuracy used as metrics. To qualitatively assess the decoding performance, we select some random images and visualize the retrieved nearest neighbors in image space based on the predicted CLIP embeddings with our model. For each test image, we display the original stimulus alongside its top-5 neighbors retrieved from the test set. Figure 3 illustrates the qualitative retrieval results obtained using our proposed model.

Table 1 reports the retrieval accuracy across different decoding models and feature processing strategies. Our best-performing model combines temporal attention with a shallow MLP, achieving the highest retrieval performance (69.3% top-1 / 93.6% top-5) and outperforming both linear baselines and more complex models such as LSTMs.

**Table 1.** Retrieval performance averaged over five seeds and two primates with different decoding models (using all channels).

Decoding Model	Top-1 Accuracy		Top-5 Accuracy	
	MSE Loss	NT-Xent Loss	MSE Loss	NT-Xent Loss
Linear/TimeFlat	10.1% ± 2.10%	41.3% ± 3.92%	27.3% ± 2.89%	72.6% ± 5.66%
Linear/AvgTime	21.4% ± 1.36%	54.9% ± 1.53%	45.0% ± 2.97%	85.1% ± 1.67%
MLP/TimeFlat	12.6% ± 1.88%	47.7% ± 2.38%	35.3% ± 2.60%	78.7% ± 1.99%
MLP/AvgTime	24.0% ± 1.10%	66.1% ± 2.08%	50.6% ± 2.42%	90.1% ± 2.48%
LSTM	20.0% ± 2.38%	63.3% ± 3.48%	45.9% ± 3.02%	90.6% ± 2.89%
TCN	18.1% ± 2.51%	59.1% ± 3.01%	44.1% ± 2.56%	86.6% ± 1.77%
Linear/TimeAtt	19.4% ± 3.11%	62.5% ± 2.89%	42.8% ± 2.70%	89.1% ± 1.56%
MLP/TimeAtt	22.4% ± 3.14%	**69.3%** ± **2.38%**	47.8% ± 3.54%	**93.6%** ± **1.03%**

Check Table S1 in Supplementary Material for statistical evidence. For completeness, we also report in Supplementary Table S2 the raw embedding prediction quality against retrieval performance for the best models.

Best results in bold.

### 3.2 Scaling laws

**Fig. 4.**
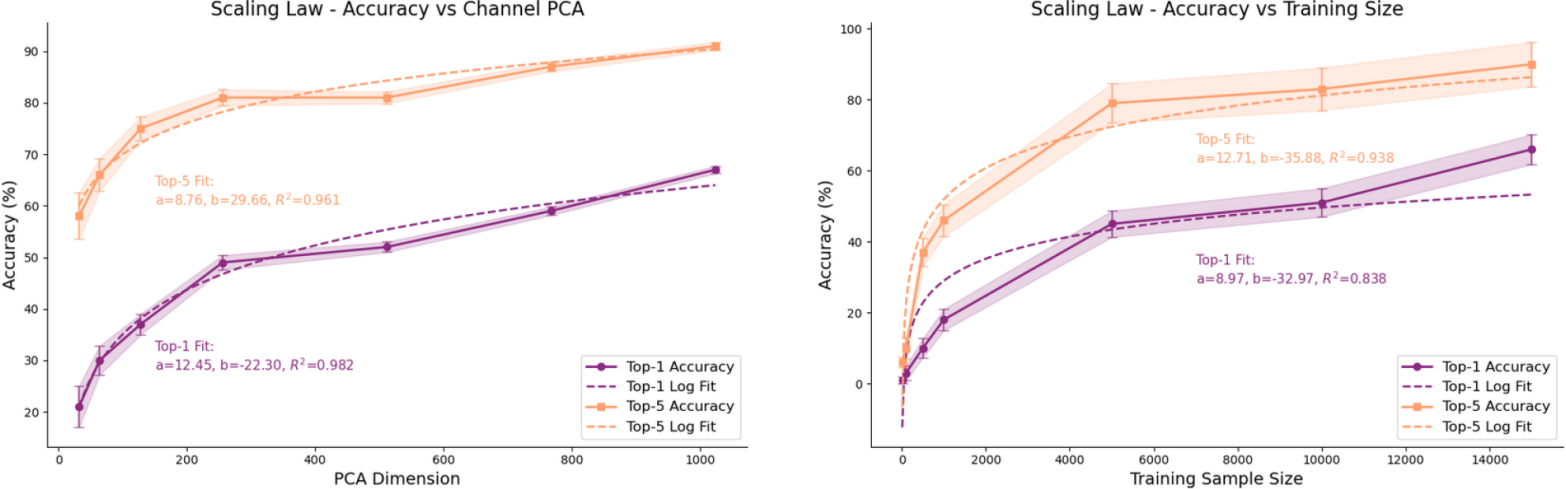
Left: Top-1 and Top-5 classification accuracy as a function of PCA dimensionality applied to neural channels. Accuracy increases logarithmically with dimensionality, as shown by high R ^2^ in the log-fit curves. Right: Top-1 and Top-5 accuracy as a function of training set size. Performance scales log-linearly with data, underscoring the importance of dataset size in brain-based visual decoding.

We investigated how model performance scales with neural feature dimensionality and the size of the training dataset. As illustrated in Figure 4 (left), increasing the number of principal components retained after applying PCA to the neural data consistently improves top-1 and top-5 classification accuracy. The trends are approximately logarithmic, as confirmed by a log-fit model, with large initial gains followed by diminishing returns beyond 256 dimensions. PCA was fitted only on training data: at 256 components, 60.1% of the total variance (starting from 35.1% with 32 components) in the neural signal is retained, suggesting that decoding information is concentrated in a relatively low-dimensional subspace of the full 1,024-channel recording. This supports the idea that while semantic information spans a high-dimensional neural space, a low-rank subspace can capture most of the information required for coarse-grained decoding. On the right (Fig. 4), we observe a strong scaling trend with respect to training set size. Performance improves rapidly as the number of training examples increases, particularly between 100 and 5,000 samples. Although gains persist beyond 10,000 samples, they begin to taper off, indicating a regime of diminishing returns. Both plots exhibit classic scaling law behavior (Antonello et al., 2023; Banville et al., 2025; Kaplan et al., 2020; Sato et al., 2024), where more data or higher-capacity representations improve performance predictably, albeit with sublinear returns.

### 3.3 Attention weights

**Fig. 5.**
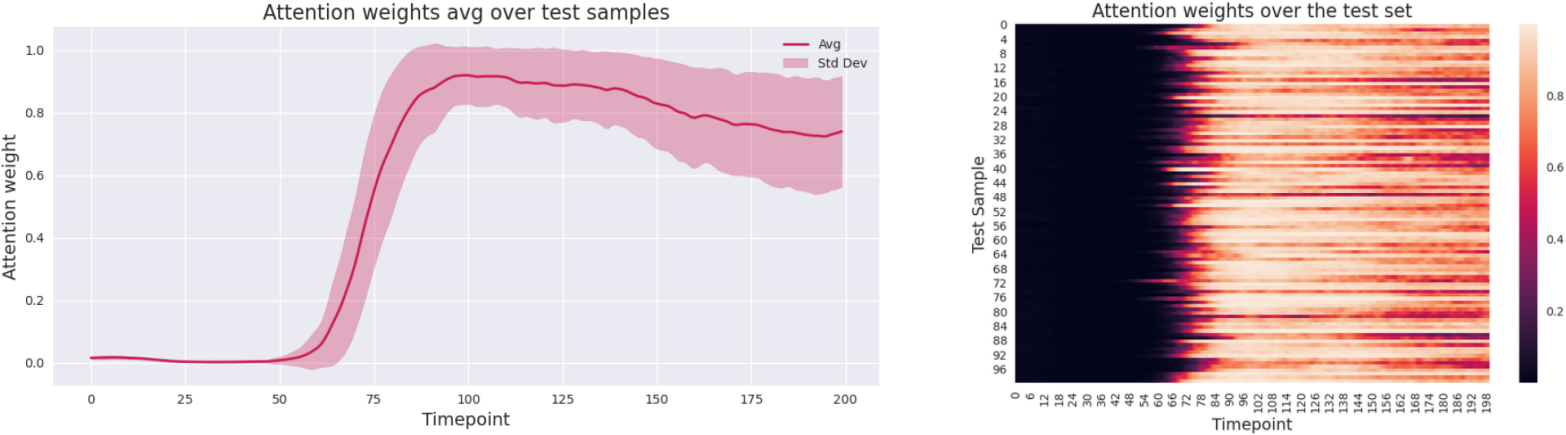
Heatmap of attention weights for the test set (right side) and average weights plot over the entire set (left side). Warmer colors of the heatmap indicate higher attention weights.

In order to gain insight into the temporal focus of the decoding model, we extracted and visualized the attention weights produced by our neural model over the entire test set. For each input sample $x\in {\mathbb{R}}^{T\times C}$ , the model outputs a sequence of attention scores α∈ℝ*^T^* reflecting the relative importance of each timepoint in the final prediction. During inference, we aggregated the attention weights for all test samples and constructed a matrix $A\in {\mathbb{R}}^{N\times T}$ , where each row corresponds to a test trial and each column to a timepoint. The attention analysis reveals interpretable patterns in the model’s temporal sensitivity (see Fig. 5).

Averaged across trials, attention weights follow a consistent rise: they remain near zero during the first 50 ms post-stimulus onset, corresponding to the afferent conduction delay before visual responses reach cortex, then rise steeply between approximately 50 and 75 ms, and plateau at high values through the remainder of the 200 ms window. The onset timing and shape of this profile are consistent with the visually evoked MUA responses documented in Papale et al. (2025) for the same dataset, where V1, V4, and IT exhibit responses with partially overlapping but staggered temporal profiles. Since the full-model attention is computed over channels from all three areas jointly, the learned weights can be understood as a data-driven, task-optimized integration of these area-specific temporal envelopes—earlier-responding channels (predominantly V1) contributing to the rising phase, and later, more sustained responses (V4 and IT) sustaining the plateau. The lightweight temporal selection module assigns data-driven weights to each millisecond of multi-unit activity, and this selective temporal integration provides a consistent benefit over uniform averaging, without requiring complex recurrent or convolutional temporal modeling. We also report in Supplementary Figure S2 the retrieval performance at millisecond level, implementing a sliding estimator.

### 3.4 Image reconstruction

We evaluated the generative potential of our brain decoding model by estimating the latent representations required to guide the pretrained diffusion model. We adopt a semantic-first rejection sampling strategy, in which multiple candidate images are generated from the predicted embedding and ranked according to their structural consistency with a low-resolution preview (using the VAE component of the model) decoded from brain activity.

**Fig. 6.**
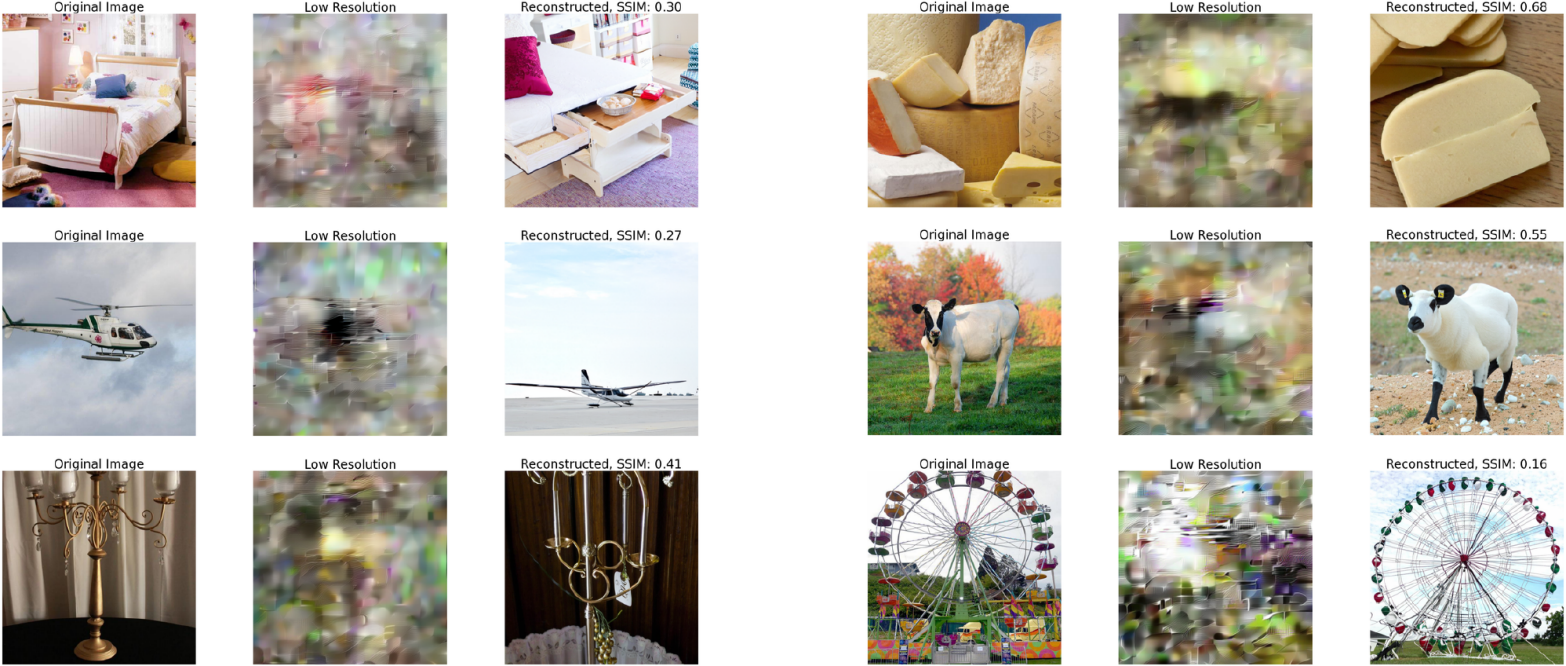
Examples of image reconstructions from neural activity. Each triplet shows the original image (left), a low-resolution baseline (middle), and our reconstruction (right) with corresponding SSIM score. The generations capture essential attributes such as object structure, color distribution, and general content.

Figure 6 shows representative examples of the image generation results. For each test sample, we display the original stimulus, the brain-inferred low-resolution preview, and the high-resolution reconstruction generated by the diffusion model conditioned on the estimated visual embedding. Reconstructions are ranked on the basis of the structural similarity index (SSIM).

Table 2 reports the evaluation metrics in image reconstruction for different model configurations. Across all metrics, the variants exhibit similar performance, with subtle differences depending on the evaluation criterion. MLP with time attention shows better results in perceptual and semantic metrics (e.g., CLIP 0.892 and PixCorr 0.186), indicating stronger high-level feature alignment. These results underscore the importance of evaluating reconstruction not just with pixel-wise losses but also with perceptual and embedding-based metrics, which better reflect the semantic fidelity of the decoded stimuli.

**Table 2.** Quantitative evaluation of all decoder variants.

Metric	Lin-TAtt	Lin-Avg	Lin-Flat	MLP-Flat	MLP-TAtt	MLP-Avg	TCN	LSTM
PixCorr ↑	0.140 ± 0.163	0.127 ± 0.113	0.111 ± 0.128	0.123 ± 0.109	**0.186** ± **0.160**	0.186 ± 0.163	0.099 ± 0.109	0.178 ± 0.162
SSIM ↑	0.364 ± 0.199	0.350 ± 0.182	0.324 ± 0.206	0.339 ± 0.219	0.371 ± 0.201	0.342 ± 0.206	**0.427** ± **0.190**	0.367 ± 0.203
MSE ↓	0.110 ± 0.008	0.120 ± 0.008	0.130 ± 0.006	0.128 ± 0.004	0.112 ± 0.001	**0.107** ± **0.001**	0.143 ± 0.002	0.107 ± 0.004
Cosine ↑	0.821 ± 0.108	0.810 ± 0.099	0.800 ± 0.113	0.806 ± 0.129	0.818 ± 0.111	0.801 ± 0.100	**0.846** ± **0.070**	0.819 ± 0.099
AlexNet2 ↑	**0.888** ± **0.161**	0.867 ± 0.167	0.850 ± 0.158	0.859 ± 0.144	0.873 ± 0.185	0.876 ± 0.168	0.541 ± 0.285	0.874 ± 0.178
AlexNet5 ↑	0.954 ± 0.079	0.921 ± 0.093	0.905 ± 0.094	0.912 ± 0.099	**0.967** ± **0.050**	0.950 ± 0.075	0.519 ± 0.277	0.937 ± 0.117
Incep.V3 ↑	0.868 ± 0.225	0.842 ± 0.213	0.833 ± 0.212	0.845 ± 0.208	**0.874** ± **0.206**	0.808 ± 0.230	0.485 ± 0.284	0.827 ± 0.244
CLIP ↑	0.879 ± 0.201	0.839 ± 0.195	0.823 ± 0.219	0.839 ± 0.198	**0.892** ± **0.196**	0.827 ± 0.245	0.491 ± 0.281	0.853 ± 0.226
EffNet ↓	0.792 ± 0.144	0.822 ± 0.177	0.832 ± 0.123	0.821 ± 0.108	**0.780** ± **0.144**	0.827 ± 0.137	0.986 ± 0.045	0.805 ± 0.167
SwAV ↓	**0.492** ± **0.111**	0.512 ± 0.123	0.537 ± 0.121	0.522 ± 0.108	0.492 ± 0.112	0.528 ± 0.117	0.697 ± 0.075	0.506 ± 0.113

We also report in Supplementary Table S4 our best model performance using a different rejection-sampling criteria, a structural baseline and a shuffled embeddings control.

↑ indicates higher is better, ↓ lower is better. Best values per row are in bold.

### 3.5 ROI-wise analysis

To better understand how different regions of the ventral visual stream contribute to decoding, we performed a set of supplementary analyses in which the proposed MLP/TimeAtt model was evaluated separately on channels belonging to V1, V4, and IT, as well as under a leave-one-ROI-out setting where one region was removed at a time (see also Figure S5 in Supplementary Material). In addition, we inspected the temporal attention profiles learned when the model was trained on each ROI independently, and when one ROI was excluded from the input.

**Fig. 7.**
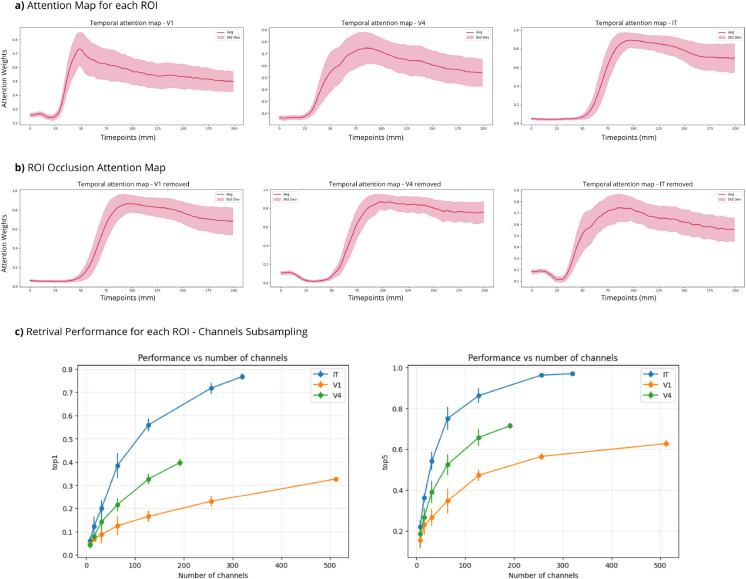
ROI-wise analyses. (a) Temporal attention maps learned when the model is trained on V1, V4, or IT channels only. (b) Attention maps in the leave-one-ROI-out setting, where one region is removed at a time. (c) Retrieval accuracy as a function of the number of channels for each ROI. For each point, channels are randomly subsampled and the experiment is repeated 30 times, reporting the mean performance.

Figure 7 summarizes three complementary observations. First, ROI-specific attention maps reveal distinct temporal profiles across visual areas. V1 exhibits an earlier rise in attention, consistent with its role in early visual processing, whereas V4 and especially IT show later and more sustained temporal weighting. Second, occlusion analyses indicate that removing a single ROI changes the temporal allocation of the model, suggesting partial compensation by the remaining areas. Third, retrieval performance as a function of channel count highlights substantial differences in information content across ROIs: IT consistently provides the strongest decoding signal, followed by V4, while V1 contributes less when used in isolation.

These trends are confirmed quantitatively in Table 3. Together, these results suggest that high-level ventral stream areas carry the dominant semantic information required for retrieval, while earlier areas provide a weaker but still partially complementary contribution.

**Table 3.** Retrieval performance of the proposed decoder across ROI-specific and leave-one-ROI-out settings (mean ± standard deviation).

Setting	Top-1 Accuracy	Top-5 Accuracy
V1 retrieval	24.4 ± 1.8	58.3 ± 1.5
V4 retrieval	33.1 ± 2.0	66.7 ± 1.6
IT retrieval	67.5 ± 2.1	91.2 ± 1.2
No-V1 retrieval	68.1 ± 2.2	92.1 ± 1.4
No-V4 retrieval	67.2 ± 2.3	88.9 ± 1.5
No-IT retrieval	44.9 ± 2.6	73.7 ± 1.9
Full ROIs	69.3 ± 2.4	93.6 ± 1.0

IT is the most informative region when evaluated independently, while excluding IT produces the largest degradation in performance.

## 4 Discussion

We investigated which factors influence visual decoding accuracy from primate intracortical recordings the most. We focused on temporal dynamics, model complexity, loss functions, and data scaling. First, we show that a compact temporal selection module can turn noisy, prone-to-overfitting neural activity into a stable semantic representation that directly supports zero-shot identification of the viewed stimulus. Second, we turn that semantic representation into plausible reconstructions using a modular generation pipeline that explicitly separates semantic identity from structural layout via rejection sampling. Beyond model comparison, the ROI-wise analysis provides a direct window into the regional organization of visual information in the macaque ventral stream. In the following, we summarize our key findings and their broader implications.

### 4.1 Non-linearity and selective temporal integration improve decoding

We find that two factors jointly contribute to decoding performance. First, introducing nonlinearity (MLP over linear projection) produces a clear gain, under NT-Xent loss. Second, replacing uniform averaging with selective temporal attention yields a consistent and significant improvement on top of this nonlinear baseline. Notably, heavier sequence models (TCN and LSTM) do not consistently outperform MLP/AvgTime and remain below MLP/TimeAtt overall, despite their greater capacity for modeling temporal dependencies. High-resolution neural data contain many low-informative timepoints; sequence models propagate all of them, which increases noise sensitivity. In contrast, our soft-attention mechanism learns to upweight the most informative intervals, effectively filtering out noisy or irrelevant fluctuations.

### 4.2 Retrieval-based evaluation improves interpretability

Throughout our analyses, we prioritized retrieval-based evaluation over direct image generation as a metric of decoding success. By mapping neural activity to a fixed, semantically grounded embedding space (CLIP), we bypassed the confounding influence of pixel-level generative priors that often obscure whether high-quality outputs reflect accurate brain decoding (Shirakawa et al., 2024). However, we acknowledge that this substitution does not eliminate priors, it redistributes them. CLIP embeddings are themselves a powerful prior, shaped by large-scale human-labelled image-text data, and high retrieval accuracy reflects alignment between primate ventral stream activity and a *human-derived* semantic space rather than a prior-free mapping from neural activity to perceptual content. Our claim is not about prior-free decoding, but a choice to make the nature and point of entry of priors explicit and controllable.

### 4.3 Scaling laws reveal predictable tradeoffs

Investigating the scaling behavior revealed consistent trends (Antonello et al., 2023; Banville et al., 2025). Increasing the input dimensionality by retaining more principal components from the PCA decomposition led to substantial improvements in decoding accuracy, but with diminishing returns beyond approximately 256 components, suggesting that a relatively low-dimensional neural manifold suffices for capturing most semantic information. Similarly, scaling the number of training trials sharply improved performance, with top-5 retrieval accuracy increasing up to 90% as the training set approached 15,000 examples. Together, these scaling laws provide practical guidance for experimental design: to optimize decoding, the most effective thing is to acquire more diverse trials. Increasing input dimensionality—such as through higher channel count—also contributes to performance gains. Next-generation BCI systems could prioritize large-scale data collection and ultra-high-density recordings to further enhance decoding accuracy.

### 4.4 Contrastive loss vs MSE loss

Across multiple decoding architectures, NT-Xent-trained models consistently achieve higher Top-1 and Top-5 accuracy. Beyond empirical performance, the outcome is also grounded in theoretical work: recent findings (Piantadosi et al., 2024) argue that conceptual and semantic representations are best modeled as directional vectors in high-dimensional spaces. In this view, the direction of a vector encodes most of the meaningful structure, making cosine similarity—sensitive to direction but not to scale—particularly well-suited for tasks involving semantic embeddings such as ours.

### 4.5 Generative decoding benefits from modularity

Building on robust retrieval performance, we developed a two-stage generative decoding pipeline. Rather than directly mapping neural data to high-resolution images, we split the task into semantic candidate generation via a frozen diffusion model and low-resolution structure reconstruction via direct latent-space prediction. Rejection sampling based on structural similarity (SSIM) allowed us to combine the best elements of both streams: semantic accuracy and structural faithfulness. This modularity avoided entangling the training objective with generative biases and offers a flexible blueprint for future decoding systems capable of both recognition and synthesis. The genuine contribution of our framework lies not in removing priors, but in cleanly separating their points of entry: the semantic prior (CLIP) and the generative prior (Stable Diffusion) operate at distinct, auditable stages, each independently validatable.

### 4.6 Complementarity with existing approaches

Compared to contemporaneous efforts such as MonkeySee (Le et al., 2024), which focus on pixel-level reconstruction using CNN-based spatial mappings, our work emphasizes semantic decoding, temporal modeling, and principled evaluation through retrieval metrics. Together, these approaches are complementary: high-fidelity spatial reconstructions and semantically aligned representations represent two sides of the brain decoding challenge. Bridging them could offer a richer, multi-level understanding of neural information flow. See also Table S3 in Supplementary Material for fair comparisons.

### 4.7 Failure modes and cross-species insights

The observed reconstruction failures are scientifically informative rather than incidental. Object identity drift (e.g., a cow rendered with sheep-like features), structural conflation (e.g., a bed acquiring desk-like properties), and changes in object numerosity suggest that the decoded signal captures coarse category-level information without preserving fine-grained relational or structural detail. These failure modes are consistent with the known representational limits of a global CLIP embedding as an intermediate: by collapsing an image into a single vector, fine-grained intra-category distinctions and compositional structure are partially lost before decoding even begins. More fundamentally, many of the semantic categories (see Figure S4 and Table S5 in Supplementary Material) used in this study are not ecologically meaningful to a macaque: the conceptual distinctions between a desk and a bed, for example, are human cultural constructs absent from the monkey’s umwelt. High retrieval accuracy, therefore, reflects alignment between ventral stream activity and a human-derived semantic space, not evidence that the monkey brain encodes these distinctions in the same way a human would.

### 4.8 Broader impact

Our study contributes to the development of decoding systems with potential relevance for human brain–computer interfaces (BCIs). By combining high-density recordings with a large number of trials, we observed substantial improvements in decoding performance. Although current results are limited to few subjects and single modality, these outcomes suggest that modular and interpretable pipelines may inform future BCI design.

## 5 Limitations

Despite these promising results, several limitations warrant discussion. First, our retrieval-based evaluation is inherently constrained by the fixed candidate pool of images, which may underestimate the full expressive power of the decoded representations. Second, our semantic mappings rely on frozen CLIP embeddings, which were optimized for human visual-textual associations and may not perfectly align with primate visual representations (Xu et al., 2023). More fundamentally, the success of CLIP-based decoding does not imply that the brain encodes information in a CLIP-like format: our analyses show that vision-only models achieve competitive retrieval performance (see Supplementary Table S6 in Supplementary Material for details), suggesting that CLIP’s advantage is primarily pragmatic. Third, although our rejection-sampling strategy mitigates biases, the use of pretrained generative models still introduces priors that could inflate perceived reconstruction quality. Fourth, we cannot rule out the possibility that more complex, nonlinear architectures might achieve higher decoding performance. Finally, the passive viewing task minimized behavioral confounds but precluded the study of top-down modulations such as attention or memory, which could significantly shape neural dynamics during naturalistic behavior. Together, these limitations suggest fruitful directions for future research aiming to develop more robust, flexible, and biologically grounded decoding systems. Looking ahead, applications in humans raise critical concerns about neural privacy and consent (Yuste et al., 2017), calling for ethical considerations alongside technical progress.

## 6 Conclusions

Our findings show that lightweight models combining nonlinear projection with selective temporal integration can accurately decode semantics from invasive primate recordings. While nonlinearity accounts for the larger share of the performance gain, temporal attention provides a consistent benefit over uniform averaging, and proves more effective than heavier recurrent alternatives in this data regime. A modular retrieval-generative framework supports flexible and interpretable reconstructions, paving the way for future extensions to cross-subject generalization, complex behaviors, and multisensory data.

## Supplementary Material

Supplementary Material

## Data Availability

All data are from a publicly available dataset (THINGS Ventral Stream Spiking Dataset) at: https://gin.g-node.org/paolo_papale/TVSD. Implementation code for reproducibility is available at the repository: https://github.com/fidelioc55/primates-mua-decode.
