# Supplementary material for: A modular semantic-structural pipeline for visual decoding from primate spiking data via selective temporal integration
Source: Imaging Neurosci (Camb). 2026 Jul 13;4:IMAG.a.1299. doi: 10.1162/IMAG.a.1299 (PMC13366611; doi:10.1162/IMAG.a.1299)
Supplement: Supplementary Material [file IMAG.a.1299_supp.pdf]

Supplemental Material

This section provides additional qualitative and quantitative results to support the main findings of the article. Firstly, we report in Table S1 the statistical evidence in decoding results, comparing the different models tested. All models significantly exceed chance-level decoding, as confirmed by a permutation test in which training pairs were randomly shuffled (null Top-1: 0.99% ± 0.99%, null Top-5: 4.94% ± 1.92%; p value $<$ 0.001).

Metric	Comparison	Δ mean	p_{corr}	Cohen's d_z
	Primary comparisons: MLP/TimeAtt vs. closest competitors			
Top-1	MLP/TimeAtt > MLP/AvgTime	+3.2	1.1×10^{-4}	1.81
Top-1	MLP/TimeAtt > LSTM	+6.0	5.8×10^{-5}	2.27
Top-1	MLP/TimeAtt > Linear/TimeAtt	+6.8	$< 10^{-6}$	5.03
Top-5	MLP/TimeAtt > LSTM	+3.0	3.9×10^{-3}	1.24
Top-5	MLP/TimeAtt > MLP/AvgTime	+3.5	4.9×10^{-4}	1.71
Top-5	MLP/TimeAtt > TCN	+6.9	$< 10^{-6}$	5.70
	Secondary comparisons among non-best models			
Top-1	MLP/AvgTime > LSTM	+2.8	3.0×10^{-2}	0.82
Top-1	LSTM > TCN	+4.3	9.9×10^{-4}	1.54
Top-1	Linear/TimeAtt > TCN	+3.4	1.1×10^{-5}	1.89
Top-1	LSTM vs. Linear/TimeAtt	+0.8	n.s.	0.24
Top-5	LSTM > TCN	+4.0	2.8×10^{-3}	1.35
Top-5	MLP/AvgTime vs. Linear/TimeAtt	+0.9	n.s.	0.61
Top-5	LSTM vs. Linear/TimeAtt	+1.3	n.s.	0.39
Top-5	MLP/AvgTime vs. LSTM	-0.4	n.s.	-0.11

Table S1: Corrected pairwise statistical comparisons for decoding performance. The upper block highlights that *MLP/TimeAtt*, our best-performing model, significantly outperforms all relevant competitors, including the strongest baselines (MLP/AvgTime, LSTM, and TCN), in both Top-1 and Top-5 retrieval. The lower block reports the closest comparisons among the remaining models, emphasizing the relative positioning of TCN and LSTM. Δ mean denotes the mean paired performance difference (in percentage points), and corrected p-values were obtained with FDR correction.

We also report direct embedding prediction metrics for the best models (S2), in order to complete the evaluation with retrieval and generation stages. While direct embedding metrics such as cosine similarity and MSE measure pointwise alignment between predicted and target vectors, they do not capture whether a prediction is discriminative, that is, whether it is more similar to its correct target than to other images in the dataset. Retrieval accuracy directly operationalizes discriminability by asking whether the decoded embedding ranks its correct match above 99 distractors, and is therefore a more faithful measure of decoding quality in the closed-set setting used here.

Table S2: Double-stage evaluation of decoding models. Stage (i) reports raw embedding prediction quality; Stage (ii) reports closed-set retrieval accuracy.

**Model**	**Stage (i) — Embedding quality**		**Stage (ii) — Retrieval**	
	Cosine sim ↑	MSE ↓	Top-1 ↑	Top-5 ↑
MLP/TimeAtt (ours)	0.190 ± 0.005	0.286 ± 0.005	$69.3 \pm 2.4\%$	$93.6 \pm 1.0\%$
MLP/AvgTime	0.163 ± 0.004	0.467 ± 0.020	$66.1 \pm 2.1\%$	$90.1 \pm 2.5\%$
Linear/TimeAtt	0.203 ± 0.005	0.229 ± 0.001	$62.5 \pm 2.9\%$	$89.1 \pm 1.6\%$
LSTM	0.196 ± 0.007	0.333 ± 0.002	$63.3 \pm 3.5\%$	$90.6 \pm 2.9\%$
TCN	0.179 ± 0.007	1.515 ± 0.301	$59.1 \pm 3.0\%$	$86.6 \pm 1.8\%$

S1 MonkeySee Baseline

Table S3 compares feature correlations across AlexNet layers between our approach (MLP/TimeAtt with other decoders) and the MonkeySee baselines (Le et al., 2024). Our model consistently outperforms both MonkeySee Spatial and Spatiotemporal in the early convolutional layers (conv1 and conv2). This suggests that the rejection sampling procedure, which selects generated images based on low-level structural similarity from a pool of candidates, effectively enhances alignment with early visual features encoded in the brain. In the deeper fully connected layers (FC6–FC8), our model maintains strong performance, surpassing MonkeySee. This indicates that the generative decoder is also capable of capturing high-level semantic content, highlighting the model's ability to represent both perceptual structure and abstract semantics from neural activity.

**Layer**	**MonkeySee Spatial**	**MonkeySee ST**	**MLP/Att**	**MLP/Avg**	**Lin/Att**	**TCN**	**LSTM**
conv1	0.358	0.372	0.528	0.492	0.507	0.555	0.495
conv2	0.320	0.334	0.368	0.336	0.348	0.351	0.348
conv3	0.429	0.443	0.335	0.302	0.308	0.299	0.308
conv4	0.385	0.401	0.319	0.294	0.293	0.247	0.294
conv5	0.292	0.318	0.305	0.280	0.280	0.185	0.284
FC6	0.344	0.377	0.522	0.507	0.481	0.224	0.494
FC7	0.534	0.579	0.500	0.493	0.459	0.151	0.474
FC8	0.579	0.610	0.712	0.710	0.687	0.386	0.690

Table S3: Feature correlation (mean Pearson) between AlexNet features extracted from reconstructed and original images. MLP/TimeAtt achieves the best performance in higher-level representations (FC6–FC8), while TCN performs better in early convolutional layers.

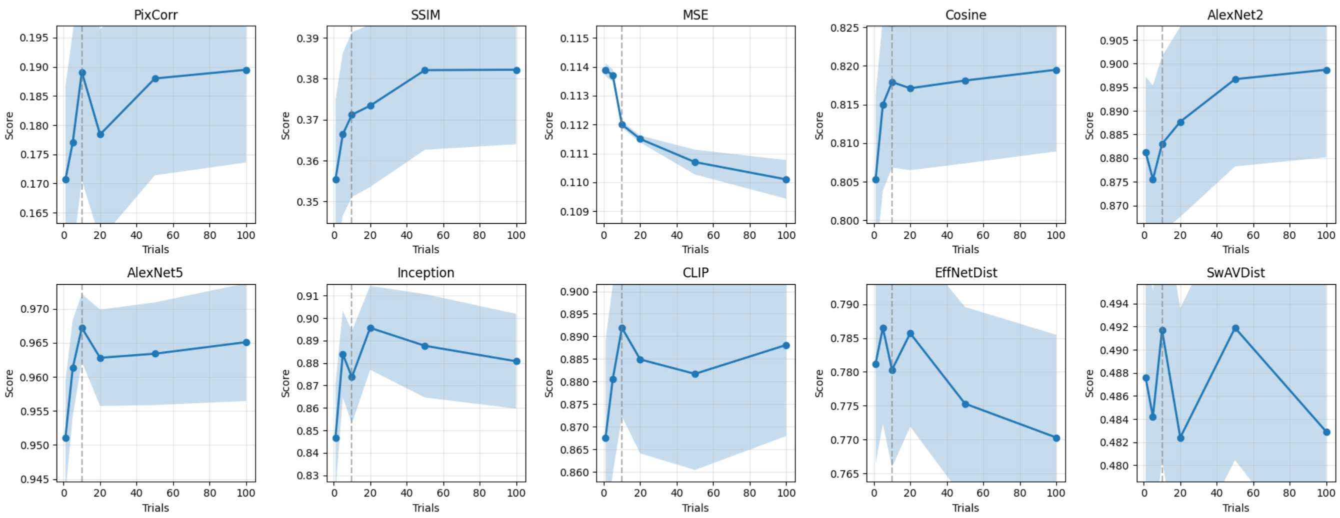

Figure S1: Ablation of rejection sampling as a function of the number of generated candidates for the best decoding model. Each panel reports the mean performance under a different evaluation metric when the final reconstruction is selected from progressively larger candidate pools. The dashed vertical line marks the best candidate count selected for reconstructions (n=10). Increasing the number of candidates generally improves the selected reconstruction, although gains are metric-dependent and tend to saturate beyond a moderate pool size.

S2 Ablation on Rejection Sampling

We conducted a set of controlled ablations that isolate different aspects of the sampling procedure and disentangle the contribution of semantic and structural information. First, we evaluated a single-candidate baseline, in which only the first generated sample is used without any ranking. Second, we considered a subset selection regime within [5, 10, 20, 50, 100] samples, where the number of candidates is progressively augmented (Figure S1). Third, we varied the ranking criterion used for selection, replacing SSIM with alternative metrics capturing different aspects of similarity, such as pixel correlation and structural features extracted from AlexNet early convolutional layers. In addition, we included two control baselines. In the shuffled-embeddings baseline, the predicted semantic embeddings are randomly permuted across trials before generation, while keeping the remaining pipeline unchanged. This control tests whether the generated images depend on trial-specific semantic neural information rather than on the generative prior and structural ranking alone. In the structural-only baseline, semantic conditioning is removed and reconstructions are generated using only the structural branch, providing an estimate of how much low-level image structure can be recovered without semantic guidance. Table S4 reports the results for our best model across different ranking criteria and control baselines.

These findings suggest that rejection sampling acts as a flexible mechanism for trading off between

Metric	SSIM ranking	AlexNet ranking	PixCorr ranking	Shuffled emb.	Structural-only
PixCorr ↑	0.186 ± 0.185	0.157 ± 0.165	0.220 ± 0.161	0.067 ± 0.141	0.294 ± 0.148
SSIM ↑	0.371 ± 0.201	0.361 ± 0.199	0.376 ± 0.193	0.316 ± 0.165	0.257 ± 0.103
MSE ↓	0.112 ± 0.002	0.112 ± 0.001	0.106 ± 0.004	0.147 ± 0.053	0.148 ± 0.025
Cosine ↑	0.818 ± 0.111	0.810 ± 0.110	0.826 ± 0.103	0.727 ± 0.101	0.774 ± 0.075
AlexNet2 ↑	0.873 ± 0.185	0.887 ± 0.165	0.887 ± 0.175	0.560 ± 0.273	0.736 ± 0.240
AlexNet5 ↑	0.967 ± 0.050	0.957 ± 0.076	0.962 ± 0.066	0.532 ± 0.300	0.650 ± 0.272
Incep.V3 ↑	0.874 ± 0.206	0.864 ± 0.221	0.889 ± 0.202	0.527 ± 0.302	0.545 ± 0.284
CLIP ↑	0.892 ± 0.196	0.848 ± 0.241	0.871 ± 0.205	0.502 ± 0.273	0.516 ± 0.292
EffNet ↓	0.780 ± 0.144	0.786 ± 0.154	0.778 ± 0.147	0.984 ± 0.043	0.979 ± 0.055
SwAV ↓	0.492 ± 0.112	0.490 ± 0.119	0.489 ± 0.116	0.702 ± 0.083	0.731 ± 0.064

Table S4: Ablation of rejection-sampling ranking criteria for our best generative decoder, compared against two control conditions. We compare candidate selection based on SSIM, an AlexNet structural score, and pixel correlation. Different ranking criteria bias the final reconstruction toward different properties: SSIM and AlexNet-based ranking favor structural/perceptual consistency, whereas pixel-correlation ranking yields the strongest overall low-level fidelity. We additionally include a *shuffled embeddings* control, where predicted semantic embeddings are permuted across trials to assess whether reconstructions truly depend on semantic neural information, and a *structural-only* baseline, where reconstructions rely only on VAE structural information.

structural fidelity and semantic alignment, depending on the ranking criterion. However, the relatively small differences between configurations indicate that part of the performance is already achieved by the generative model itself, and that rejection sampling provides an additional improvement.

S3 Temporal Information

We quantitatively assess how our model uses temporal structure by comparing it to a millisecond level baseline. Specifically, we implemented a sliding estimator, training a separate MLP for each timepoint (millisecond resolution). Each MLP maps the neural signal at a single timepoint to the target embedding and is evaluated independently. This simulates what decoding would look like at high temporal precision (millisecond-scale), without aggregating across time. Our main model instead learns to combine all 200 timepoints with a self-attention mechanism, assigning learned weights to each.

As in the Figure S2, our soft-attention model consistently outperforms the sliding baseline at each timepoint (best performance: around 30% Top1, and 60% Top5), and the curve of accuracy wrt timepoints follows the same behaviour of our temporal attention. This demonstrates that not just high-resolution data matters, but the ability of the model to aggregate temporal information in a data-driven way.

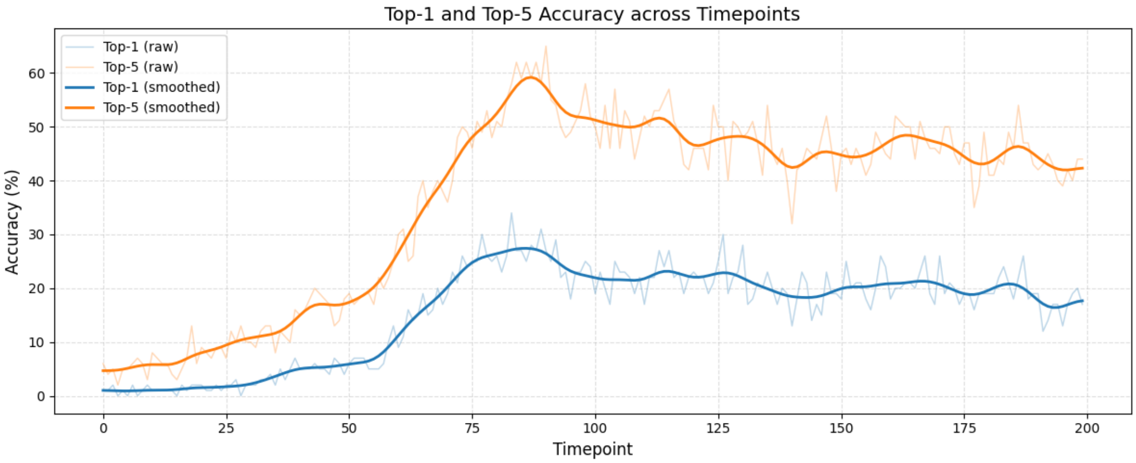

Figure S2: Decoding performance with MLP trained on a sliding estimator at millisecond level. The overall shape of performance is mirrored by our model but shifted on the y axis towards much worse performances.

S4 Neural Decoding Across Representational Spaces

A potential concern regarding our decoding framework is whether the choice of CLIP (Radford et al., 2021) embeddings as representational targets is biologically motivated, or merely an artefact of their empirical success in downstream tasks. CLIP embeddings are derived from a model trained on language-image pairs, and one might ask whether human language-aligned representations constitute the appropriate target for decoding monkey ventral stream activity.

To assess whether the categorical structure captured by CLIP is genuinely present in the neural signal, we trained a linear classifier on three different feature spaces using the same set of 21 semantic categories extracted from the Ventral THINGS dataset: (*i*) ground-truth CLIP embeddings of the stimuli, (*ii*) CLIP embeddings predicted from brain activity (i.e. the output of our decoder), and (*iii*) raw neural activity patterns. Results are reported in Table S5. All three spaces substantially exceeded chance performance, with a progressive decrease in accuracy that mirrors the information loss introduced at each stage of the decoding pipeline. Critically, the neural data alone support above-chance categorisation, indicating that the categorical structure exploited by CLIP is present in the neural signal *independently* of any language-alignment assumption.

Table S5: Categorical classification accuracy (21 classes) across three representational spaces. The same linear classifier was applied in each case. Chance level is 4.8% (1/21).

**Feature space**	**Classification accuracy**
CLIP (ground truth)	81.1%
CLIP predicted from brain	55.4%
Raw neural activity	32.4%
Chance	4.8%

To further disentangle the contribution of language alignment from that of general visual structure, we repeated the image-retrieval analysis using embeddings from two purely vision-trained models: DINOv2 (Oquab et al., 2024), ViT (Dosovitskiy et al., 2021) pretrained on ImageNet-21k without any language supervision, and AlexNet (Krizhevsky et al., 2012), a classical CNN included as a lower-bound reference representative of earlier-generation visual features. For DINOv2 and ViT we used the `[CLS]` token of the final layer and ℓ_2-normalised prior to retrieval. For AlexNet, features were extracted from the `fc7` layer (the penultimate fully connected layer, output dimensionality 4096). Results are reported in Table S6.

Table S6: Image retrieval performance (Top-1 and Top-5 accuracy, mean $\pm$ SD across cross-validation folds) for three embedding models. All models use our best neural decoder; only the target embedding space differs.

**Model**	**Top-1 accuracy**	**Top-5 accuracy**
CLIP (Radford et al., 2021)	69.3% $\pm$ 2.4%	93.6% $\pm$ 1.0%
DINOv2 (Oquab et al., 2024)	62.4% $\pm$ 3.1%	88.1% $\pm$ 2.4%
ViT (Dosovitskiy et al., 2021)	53.6% $\pm$ 2.2%	83.9% $\pm$ 1.9%
AlexNet (Krizhevsky et al., 2012)	43.9% $\pm$ 2.8%	75.1% $\pm$ 2.0%

DINOv2 and ViT achieve competitive retrieval performance despite having no exposure to language supervision during training. AlexNet `fc7`, trained with full supervision on ImageNet but lacking the representational depth of modern architectures, serves as an important control. The performance indicates that strong decoding is possible across multiple visual embedding spaces, including non-language-aligned models, but CLIP remains the best-performing target among those tested. We interpret this advantage pragmatically rather than biologically: CLIP may provide a more useful embedding geometry for this retrieval and generation pipeline, and it is also directly compatible with the image-conditioning interface used in our diffusion model.

S5 Hyperparameter Tuning

We performed a systematic hyperparameter sweep for each architecture using Weights & Biases. The explored ranges (Table S7) were chosen to balance model capacity and regularization, given the relatively limited dataset size and the temporal resolution of the input signals. For LSTM models, we explored a range of hidden sizes and depths to assess their ability to capture temporal dependencies without overfitting. For TCNs, kernel sizes and dilation depths were selected to vary the effective receptive field. Regularization parameters (dropout and weight decay) were included to explicitly test whether sequence models were under- or over-regularized. For all architectures, the choice of loss function emerged as the most influential factor. In particular, contrastive learning consistently shows a strong positive correlation with performance, while MSE-based training leads to a marked degradation, indicating that representation alignment plays a central role in this task. For the TCN model, architectural parameters such as kernel size and channel width have relatively low importance, suggesting that performance is robust to variations in receptive field and model capacity. Learning rate and MLP hidden size show moderate influence, but no strong sensitivity is observed, indicating stable optimization across configurations. In contrast, the LSTM model exhibits higher sensitivity to regularization and architectural choices. Weight decay shows a strong positive correlation with validation performance, while dropout parameters (especially in the MLP head) display negative correlation when too large, suggesting over-regularization effects. The choice of pooling strategy also impacts performance, with mean- and attention-based pooling consistently outperforming last-step aggregation. Interestingly, the number of layers and hidden dimensionality have limited influence, indicating that increasing model capacity alone does not improve generalization. Overall, these results (Figure S3) suggest that LSTM performance is primarily constrained by optimization and regularization dynamics rather than insufficient hyperparameter tuning. The broad hyperparameter sweep (including multiple forms of regularization) and the observed sensitivity patterns confirm that the lower performance of LSTM models is not due to under-tuning for this data regime.

For models trained with NT-Xent loss, the temperature parameter τ was treated as a learnable scalar initialized at 0.05 and optimized jointly with model parameters. Batch size was selected from [64, 128, 256], with 128 yielding the best validation performance.

S6 Analysis of Generative Decoding Performance

We provide two complementary analyses that characterize reconstruction quality across semantic categories, visual stimulus properties, and cortical regions of interest. Figure S4 (top) reports mean SSIM

Model	Hyperparameter	Search Space	Best
TCN - 1.3M Params.	Learning Rate	$\{1e{-}3, 1e{-}4, 1e{-}5\}$	1e-3
	Conv. Channels	$\{64, 128, 256\}$	256
	MLP Hidden Dim.	$\{128, 256, 512\}$	256
	Kernel Size	$\{3, 5, 7\}$	7
	Dropout	$\{0.0, 0.2, 0.5\}$	0.2
	Loss Type	{CL, MSE}	CL
LSTM - 4.3M Params.	Learning Rate	$\{1e{-}3, 1e{-}4, 1e{-}5\}$	1e-3
	Weight Decay	$\{0, 1e{-}5, 1e{-}4, 1e{-}3\}$	1e-3
	LSTM Hidden Dim.	$\{256, 512, 768\}$	512
	MLP Hidden Dim.	$\{256, 512, 768\}$	512
	Num. Layers	$\{1, 2, 3\}$	2
	Dropout (RNN)	$\{0.0, 0.2, 0.5\}$	0.2
	Dropout (MLP)	$\{0.0, 0.3, 0.5\}$	0.0
	Input Dropout	$\{0.0, 0.1, 0.3\}$	0.1
	Pooling	{last, mean, attn}	mean
	Loss Type	{CL MSE}	CL
Linear (TimeFlat) - 104M Params.	Learning Rate	$\{1e{-}3, 1e{-}4\}$	1e-3
	Loss Type	{CL, MSE}	CL
Linear (AvgTime/TimeAtt) - 0.5M Params.	Learning Rate	$\{1e{-}3, 1e{-}4\}$	1e-3
	Loss Type	{CL, MSE}	CL
MLP (TimeFlat) - 158M Params.	Learning Rate	$\{1e{-}3, 1e{-}4\}$	1e-3
	Num. Layers	$\{1, 2, 5\}$	2
	Hidden Dim.	$\{256, 512, 768\}$	768
	Loss Type	{CL, MSE}	CL
MLP (AvgTime/TimeAtt) - 1.2M Params.	Learning Rate	$\{1e{-}3, 1e{-}4\}$	1e-3
	Num. Layers	$\{1, 2, 5\}$	2
	Hidden Dim.	$\{256, 512, 768\}$	768
	Loss Type	{CL, MSE}	CL

Table S7: Hyperparameter search space and best configuration for each model. LSTM configurations include explicit regularization parameters (dropout and weight decay), while TCN explores architectural parameters controlling receptive field and capacity.

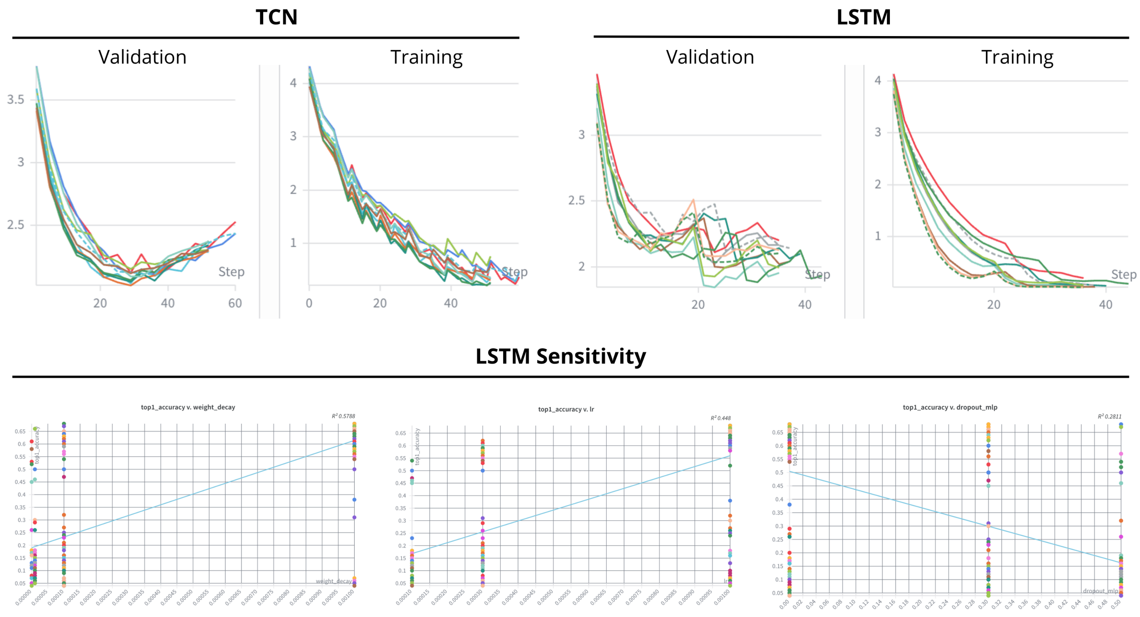

Figure S3: Training and validation loss curves (top) and LSTM hyperparameter sensitivity (bottom). LSTM shows a larger train–validation gap, indicating stronger overfitting, and higher sensitivity to optimization and regularization parameters, while TCN exhibits more stable generalization across configurations.

stratified by semantic macro-category. Reconstruction quality varies systematically with category: stimuli belonging to categories with prototypical, geometrically regular structure (such as containers, tools) yield consistently higher SSIM scores, while visually heterogeneous categories such as animals and plants score below the global mean. Notably, the animal category, which encompasses particular cases (e.g., cow/sheep identity drift), shows both below-average mean SSIM and high variance, consistent with the hypothesis that fine-grained intra-category distinctions are not fully preserved by a global CLIP embedding. We acknowledge that many categories are represented by few test samples, and results for those should be interpreted with caution.

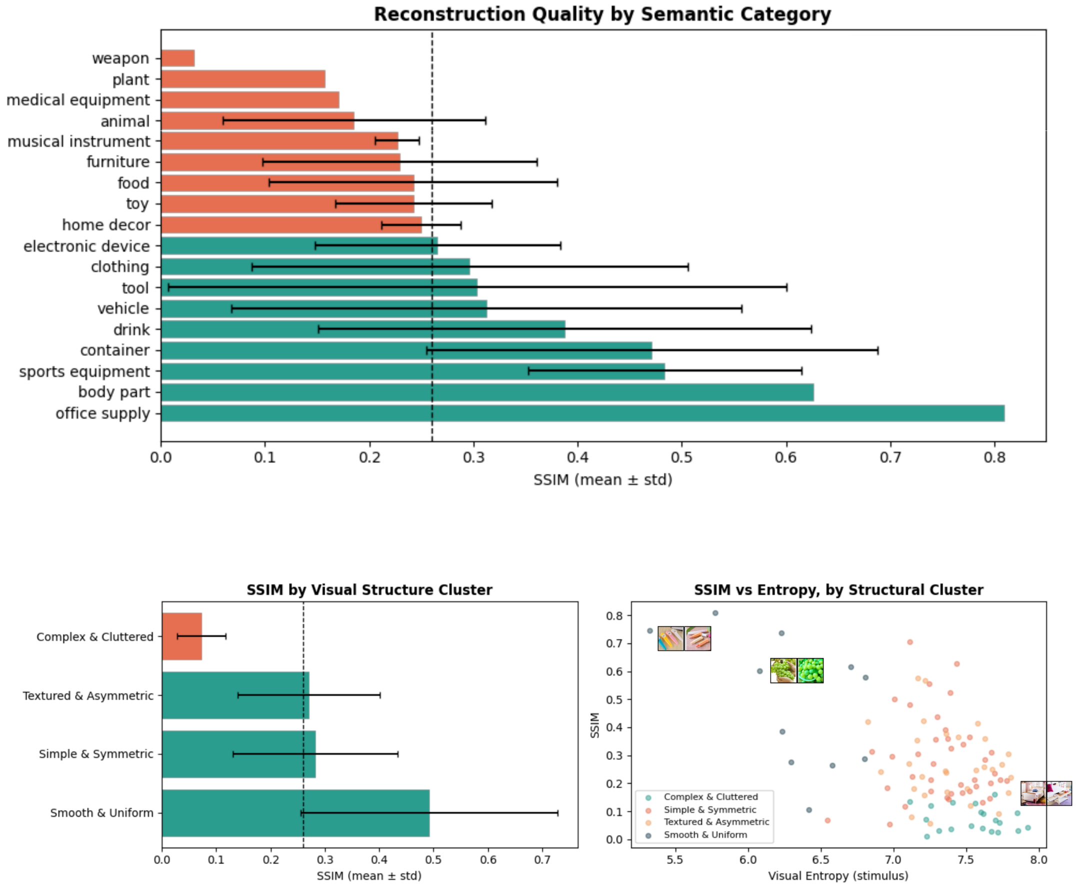

Figure S4: Reconstruction quality (SSIM) stratified by semantic macro-category (top) and by data-driven visual structure cluster (bottom). Categories with geometrically regular stimuli (e.g., drink, containers) yield higher SSIM, while visually heterogeneous categories (e.g., animals, plants) score below the global mean (dashed line). Structural clustering based on entropy, symmetry, and edge density confirms that complex, cluttered stimuli are reconstructed with significantly lower fidelity than smooth or symmetric ones.

Figure S4 (bottom) complements this view with a data-driven structural analysis. We extracted three low-level visual features from each original stimulus — Shannon entropy, horizontal symmetry, and Sobel edge density — and applied k-means clustering (k=4) to identify stimulus groups defined purely by

visual properties, independently of semantic labels. The resulting clusters, labeled Complex & Cluttered, Textured & Asymmetric, Simple & Symmetric, and Smooth & Uniform, show a clear ordering in reconstruction quality: the Complex & Cluttered cluster yields the lowest SSIM, while Smooth & Uniform stimuli show the highest mean but also the largest variance. The scatter plot of SSIM against visual entropy confirms a mild negative trend: higher stimulus complexity is associated with lower reconstruction fidelity, though with substantial residual variance. This residual variance suggests that structural complexity alone does not fully determine reconstruction quality: semantic factors and the quality of the decoded CLIP embedding play an additional role.

Figure S5 illustrates reconstructions obtained when the decoder is trained on channels from all ROIs jointly (ALL), or restricted to IT, V1, or V4 individually. Across all four stimulus examples shown, the full-ROI model consistently produces the highest-fidelity reconstructions, confirming the complementarity of ventral stream areas reported in the retrieval analysis. Restricting decoding to IT alone preserves semantic identity reasonably well. V1-only reconstructions show a marked degradation in semantic content, consistent with V1's role in encoding low-level spatial features rather than object-level representations. V4 occupies an intermediate position, preserving some structural properties, but lacking the categorical specificity of IT. These observations are qualitatively consistent with the known functional hierarchy of the ventral stream and with the quantitative retrieval results. The Ferris wheel example is particularly instructive: its radial, high-contrast structure — encoding well in both V1 and V4 spatial filters — allows even lower-area decoders to partially reconstruct the circular geometry, while semantic disambiguation (wheel vs. grid) requires IT-level representations.

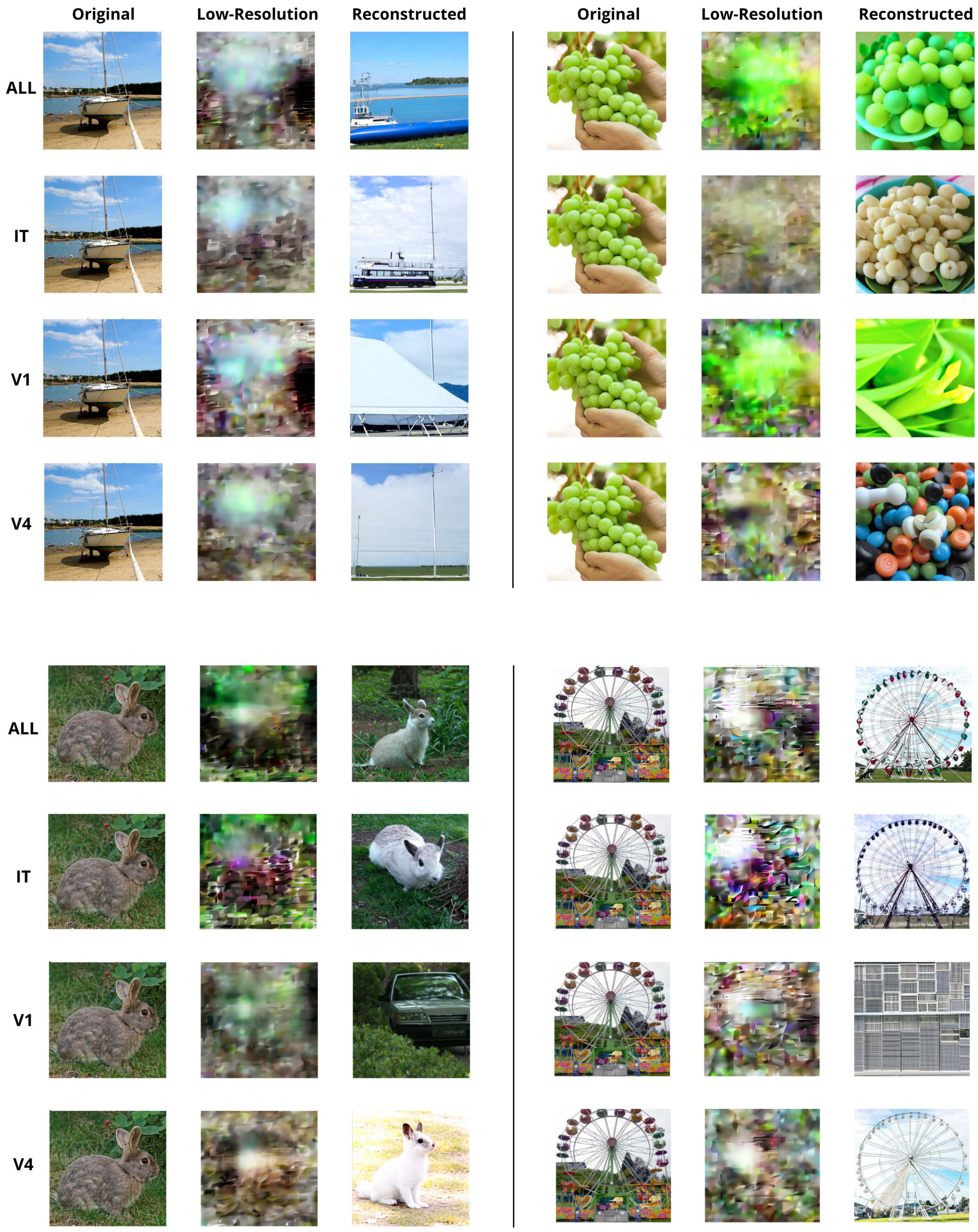

Figure S5: Four different test samples, comparing reconstruction using: all ROI's, only the IT channels, only V1 and V4 respectively.

References

Dosovitskiy, A., Beyer, L., Kolesnikov, A., Weissenborn, D., Zhai, X., Unterthiner, T., Dehghani, M., Minderer, M., Heigold, G., Gelly, S., Uszkoreit, J., & Houlsby, N. (2021). An image is worth 16x16 words: Transformers for image recognition at scale. https://arxiv.org/abs/2010.11929

Krizhevsky, A., Sutskever, I., & Hinton, G. E. (2012). Imagenet classification with deep convolutional neural networks. *Advances in neural information processing systems*, *25*.

Le, L., Papale, P., Seeliger, K., Lozano, A., Dado, T., Wang, F., Roelfsema, P., van Gerven, M., Güçlütürk, Y., & Güçlü, U. (2024). Monkeysee: Space-time-resolved reconstructions of natural images from macaque multi-unit activity. In A. Globerson, L. Mackey, D. Belgrave, A. Fan, U. Paquet, J. Tomczak, & C. Zhang (Eds.), *Advances in neural information processing systems* (pp. 93826–93848, Vol. 37). Curran Associates, Inc. https://proceedings.neurips.cc/paper_files/paper/2024/file/aa7eb65738b5bc71c81848fba9111c97-Paper-Conference.pdf

Oquab, M., Darcet, T., Moutakanni, T., Vo, H., Szafraniec, M., Khalidov, V., Fernandez, P., Haziza, D., Massa, F., El-Nouby, A., Assran, M., Ballas, N., Galuba, W., Howes, R., Huang, P.-Y., Li, S.-W., Misra, I., Rabbat, M., Sharma, V., ... Bojanowski, P. (2024). Dinov2: Learning robust visual features without supervision. https://arxiv.org/abs/2304.07193

Radford, A., Kim, J. W., Hallacy, C., Ramesh, A., Goh, G., Agarwal, S., Sastry, G., Askell, A., Mishkin, P., Clark, J., Krueger, G., & Sutskever, I. (2021). Learning transferable visual models from natural language supervision. *Proceedings of the 38th International Conference on Machine Learning*, 8748–8763.
